# Crystallization of the Transdimensional Electron Liquid

**DOI:** 10.1021/acs.nanolett.5c03894

**Published:** 2026-03-10

**Authors:** Igor V. Bondarev, Alexandra Boltasseva, Jacob B. Khurgin, Vladimir M. Shalaev

**Affiliations:** † Department of Mathematics & Physics, 3066 North Carolina Central University, Durham, North Carolina 27707, United States; ‡ Elmore Family School of Electrical & Computer Engineering, Purdue Quantum Science & Engineering Institute, and Birck Nanotechnology Center, West Lafayette, Indiana 47907, United States; ¶ School of Materials Engineering, 311308 Purdue University, West Lafayette, Indiana 47907, United States; § Department of Electrical & Computer Engineering, Whiting School of Engineering, 1466 Johns Hopkins University, Baltimore, Maryland 21218, United States

**Keywords:** Transdimensional Metals, Strong Electron Correlations, Wigner Crystal

## Abstract

Wigner crystallization of free electrons at room temperature has been explored theoretically for a new class of metallic ultrathin (transdimensional) materials whose properties can be controlled by their thickness. Our calculations of the melting surface, critical electron density and temperature explain consistently the experimental data reported previously. We show that by reducing the material thickness one can Wigner-crystallize free electrons at room temperature to get them pinned onto a two-dimensional triangular lattice of a supersolid inside of the crystalline material. Such a solid melts and freezes reversibly with increase and decrease of electron doping or temperature, whereby its resistivity behaves opposite to the free electron gas model predictions.

## Graphical abstract

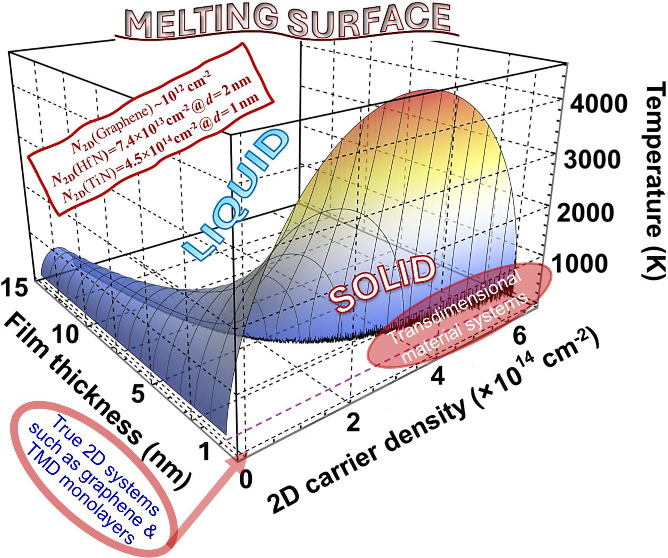

Condensed matter systems with strong electron correlations have long been in the focus of theoretical and experimental studies due to their unique physical properties.^1–12^ These studies have now evolved into a vibrant field of quantum nanomaterials to explore correlated electron systems of reduced dimensionality for remarkable phenomena such as high-temperature (*T*) quantum phase transitions,^13–15^ superconductivity,^16–18^ unconventional magnetism,^21^ and a variety of metal–insulator transitions (MITs) including quantum- and disorder-related Anderson localization,^19,20^ Kondo effect,^22,23^ Wigner crystallization^24–33^ and beyond.^34–38^ These effects are studied in various strongly correlated materials for electrons, excitons and their complexes,^38–43^ particularly in the low-dimensional regime, in systems such as semiconductor quantum wells, graphene and transition metal dichalcogenides (TMDCs).

One of the most interesting MIT phenomena is the electron Wigner crystal formation^1^—the longest anticipated exotic correlated phase of metals and metallic compounds that has intrigued physicists since 1934.^44^ In this phase free electrons crystallize in metals on a periodic lattice to form a solid made of a superlattice of electrons inside of a crystalline material. Electrons become pinned (frozen) periodically when their potential repulsion energy exceeds both the mean kinetic energy per particle and the energy of thermal fluctuations, with their density and *T* not to exceed dimension-dependent critical values.^7^ Such a solid melts and freezes up reversibly with increase and decrease of *T*, respectively, and its resistivity *T*-dependence is opposite to the free electron gas model predictions. In spite of a large body of research, achieving and observing electron Wigner crystallization remains an outstanding challenge that requires high quality, structurally stable metallic compounds with tailorable electronic response and low disorder to succeed. Thus far, signatures of electronic Wigner crystallization were observed indirectly in 2D electron gas systems under high magnetic fields^12,26^ and in twisted bilayer TMDC moiré superlattices (generalized Wigner crystals^30–36^ ). Only recently, the first microscopic images to prove charge excitations in one-dimensional (1D),^24,25^ nonzero magnetic field 2D^27^ and generalized 2D Wigner crystals were reported.^31–33^ The last two are different from the Wigner’s electron crystal concept as the ”crystallization” there is due either to magnetic localization or to moiré potential trapping of electrons instead of their Coulomb repulsion. To date, the zero-field electron crystallization has been observed indirectly in semiconducting TMDC monolayers^28^ and in untwisted homobilayers^29^ by monitoring exciton photoluminescence intensity. Specifically, an extra peak was detected that could originate from the exciton Umklapp scattering by the 2D electron lattice formed below the Wigner crystal melting point ( ∼10 K).^28^ All these studies require low *T* and external means to reduce electron mobility (magnetic field, moiré potential). Thus, the observation of the Wigner’s prediction in conventional materials remains elusive.

With current nanofabrication technology development, an exciting opportunity to study strongly correlated phenomena is offered by the so-called transdimensional (TD) material platform.^45^ Originally proposed in the field of nanoplasmonics,^46–49^ these ultrathin—between 2D and 3D—materials are expected to support strong electron correlations and could potentially enable quantum phenomena such as Wigner crystallization.^50^ Metallic and semimetallic TD compounds can have thicknesses of only a few atomic layers and show unprecedented tailorability of their electromagnetic (EM) response.^51–56^ This includes unusually strong dependence on structural parameters such as thickness (number of atomic monolayers), composition (stoichiometry, doping), strain and surface termination compared to conventional thin films, as well as extreme sensitivity to external optical and electrical stimuli. Recently, epitaxial TD films of transition metal nitrides (TMNs) such as TiN, ZrN and HfN, have been studied extensively and demonstrated their confinement-induced nonlocal EM response as well as new associated physical effects.^57–61^ However, while quite a few confinement-induced plasmonic effects have been reported for TD films experimentally,^59–62^ until very recently TD materials have not been used to explore strongly correlated electron regimes. The first experimental evidence for plasmonic behavior breakdown and related MIT was reported recently for room-*T* HfN films^60^ decreasing in thickness *d* to become a transparent dielectric at *d* = 2 nm. The unique possibility to observe the reversible MIT due to electron Wigner crystallization in vertically confined planar metallic structures not only provides insights into strong electron correlation phenomena but is also attractive for nanophotonics applications. When free electrons crystallize into a superlattice, the TD film turns into an optically transparent dielectric. When the electron solid melts, the film restores its plasmonic response. The exploration of Wigner crystallization in TD materials opens a new avenue for the realization of optical modulation and switching with this new photonic material platform.

Here, we develop a theory to generalize the Platzman-Fukyuama (PF) model of the Wigner crystal formation in free-standing 2D electron gas systems,^7^ to the practical case of TD plasmonic materials. Our calculations of the critical density, temperature, and the actual melting surface to identify the Wigner crystal phase in the available (broader) parameter space, show that TD materials offer a unique possibility. Specifically, we show that it is even possible to crystallize electrons at room *T* by simply reducing the thickness of the material. By reducing the thickness one decreases the electron density and, at the same time, enhances the interelectron repulsive potential due to the vertical confinement. Clearly, it is not only that the Coulomb repulsion is stronger for two like-charge carriers separated by the same in-plane distance but shorter out-of-plane confinement length, as it can be seen in Figure 1 (a), but also that the electrostatic field contribution they produce in lower dielectric permittivity surroundings outside of their confinement region starts enhancing their repulsion due to the reduced screening when the film thickness becomes less than the mean intercharge in-plane distance. In other words, while in bulk metals electron repulsion is screened out, in TD metals electrons can interact effectively via lower permittivity sub- and superstrates. As a result, by reducing the film thickness one can increase their repulsive potential energy to exceed the mean single-particle kinetic energy and thus to promote electron Wigner crystallization.

**Figure 1:**
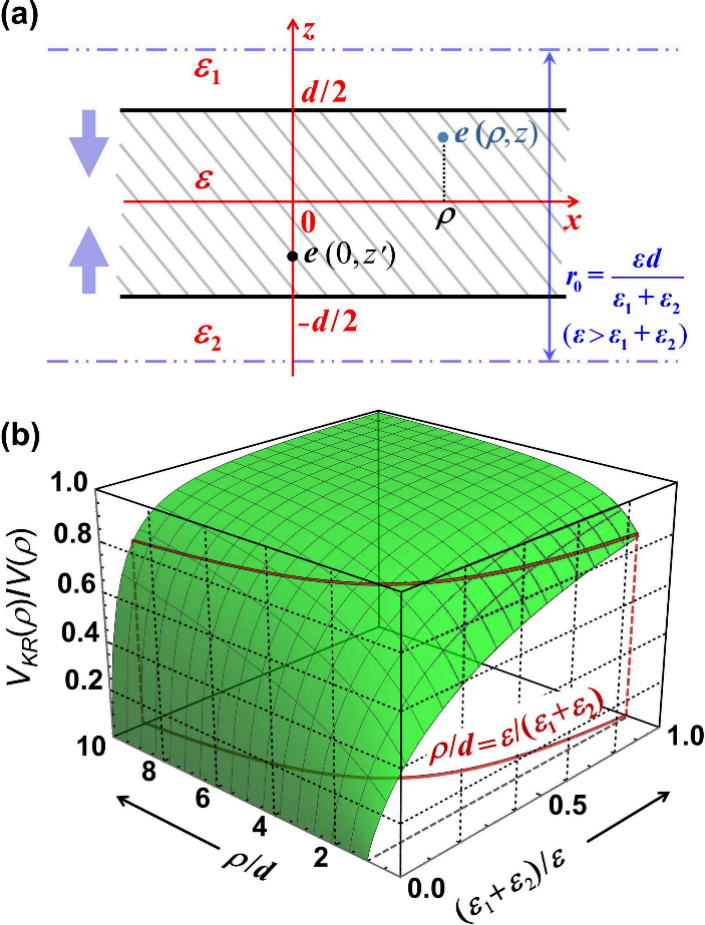
(a) Schematic to show the geometry of the KR electrostatic potential (2) for a pair of electrons confined in the ultrathin TD slab of decreasing thickness. (b) KR interaction potential (2) normalized (divided) by the 2D Coulomb interaction potential given by eqs 2 and 3 in the limit ρ ≫ *r*_0_ (or *d* → 0).

As first formulated by Platzman and Fukuyama,^7^ an ensemble of repulsively interacting particles (or quasiparticles) is expected to form a Wigner crystal lattice when its average pair potential interaction energy ⟨*V*⟩ exceeds the average kinetic energy per particle ⟨*K*⟩. Then the ratio Γ_0_ = ⟨*V*⟩/⟨*K*⟩ > 1 (referred to as the PF ratio below) represents the phase diagram (melting curve) of the process if Γ_0_ is known. The PF model describes an idealized 2D electron gas system, free-standing in air, where

$$\left\langle V\right\rangle =e^{2}/\left\langle \rho \right\rangle =e^{2}\sqrt{\pi n}$$

with *n* being the 2D electron density defined by mean in-plane interelectron distance ⟨ρ⟩ through the constraint π⟨ρ⟩^2^ = 1/*n*. While both liquids and solids are known to support longitudinal vibrational modes, transverse vibrational modes are only supported by solids and not by liquids. Hence, the transverse vibrational mode instability of the 2D electron Wigner crystal is to signal the onset of its melting. The PF model shows numerically that this is indeed the case—the transverse mode breaks down as *T* increases while the longitudinal (plasmon) mode remains quite unaffected. As such, the derivative of pressure (caused by plasma oscillations) over *T* should satisfy the well-defined Clausius–Clapeyron equation (see, e.g., ref 63) representative of the first-order “solid–liquid” phase transition (melting) in the system. The PF theory leads to Γ_0_ ≈ 3 at melting,^7^ with zero-*T* critical density scaling unit

$$n_{c}=1/\left(\pi a_{B}^{2}\Gamma_{0}^{2}\right)\approx 5\times 10^{15}\;{\mathrm{cm}}^{-2}$$

if calculated using the 2D Bohr radius *a*_*B*_ = *ℏ* ^2^/(2*me* ^2^) = 0.529/2 Å. The critical temperature scaling unit given under the classical energy equipartition by

$$\Gamma_{0}=e^{2}\sqrt{\pi n_{c}}/\left(k_{B}T_{c}\right)$$

equals

$$k_{B}T_{c}=2Ry/\Gamma_{0}^{2}\approx 6\;\mathrm{eV}$$

with the 2D Rydberg constant *Ry* = *e* ^2^/(2*a*_*B*_) = 27.25 eV.

For metallic TD films of thickness *d*, the PF ratio is

1 $$\Gamma =\frac{\left\langle V_{KR}\right\rangle }{\left\langle K\right\rangle }$$

Here

2 $$V_{KR}\left(\rho \right)=\frac{e^{2}\pi }{\left(\varepsilon_{1}+\varepsilon_{2}\right)r_{0}}\left[H_{0}\left(\frac{\rho }{r_{0}}\right)-N_{0}\left(\frac{\rho }{r_{0}}\right)\right]$$

is the repulsive Keldysh-Rytova (KR) interaction potential^64,65^ written in Gaussian units as a difference of the 0-order Struve (*H*_0_) and Neumann (*N*_0_, *aka* ^66^ Bessel *Y*_0_) special functions, where *r*_0_ = *εd*/(*ε*_1_ + *ε*_2_) = 2*πα*_2*D*_ is the screening length with α_2*D*_ representing the in-plane polarizability of 2D material.^66^ This is the electrostatic repulsive interaction energy of a pair of electrons separated by the in-plane distance ρ and confined vertically in the interior of the optically dense TD film with the positive background permittivity *ε* > *ε*_1_, *ε*_2_ of superstrate and substrate as shown in Figure 1(a). The KR potential indicates that in such optically dense ultrathin planar systems the vertical electron confinement leads to the effective dimensionality reduction from 3D to 2D, with the *z*-coordinate of the potential replaced by new parameter *d* representing the vertical size. The potential *V*_*KR*_ can be shown to go logarithmically with ρ for *d* ≪ ρ ≪ *r*_0_ and fall off as 1/ρ for ρ ≫ *r*_0_;^64,66^ see Figure 1(b). It can be accurately approximated by elementary functions as

3 $$V_{KR}\left(\rho \right)\approx \frac{2e^{2}}{\left(\varepsilon_{1}+\varepsilon_{2}\right)r_{0}}\left[\ln\left(1+\frac{r_{0}}{\rho }\right)+\left(\ln2-\gamma \right)e^{-\rho /r_{0}}\right]$$

­(γ ≈ 0.577 is the Euler-Mascheroni constant). This expression was originally proposed for monolayer semiconductors.^66^ It can be seen from eq 3 that the PF model is inappropriate for the description of the finite-thickness TD films as the standard 2D Coulomb coupling is not the case there. In the pure 2D regime (*d* → 0) where it is set to work, the PF ratio is still to be multiplied by 2/(*ε*_1_ + *ε*_2_) to include the substrate and superstrate for realistic atomically thin but optically dense materials.

The mean electron kinetic energy per particle can be calculated analytically for all *T* < *T*_*F*_, the Fermi temperature of metals ( $∼10^{5}$ K) by integrating over the 2D reciprocal space,^67^ to yield

4 $$\left\langle K\right\rangle =\frac{\pi n\hbar^{2}}{2m}\left[1+\frac{1}{3}{\left(\frac{mk_{B}T}{n\hbar^{2}}\right)}^{2}\right]=k_{B}T_{c}\;\nu \left(1+\frac{\pi^{2}t^{2}}{12\nu^{2}}\right)$$

Here, ν = *n*/*n*_*c*_ and *t* = *T*/*T*_*c*_ are the electron density and temperature, respectively, made dimensionless using the critical density and critical temperature scaling units above for convenience of comparison with the PF model.

Plugging eqs 2 and 4 in eq 1 leads to

5 $$\frac{\Gamma }{\Gamma_{0}}=\frac{F\left(d,\varepsilon ,\varepsilon_{1,2}\right)}{\nu \left[1+\pi^{2}t^{2}/\left(12\nu^{2}\right)\right]}$$

where

6 $$F\left(d,\varepsilon ,\varepsilon_{1,2}\right)=\pi \Gamma_{0}\frac{H_{0}\;\left[\Gamma_{0}/\left({\mathrm{r̅}}_{0}\sqrt{\nu }\right)\right]-N_{0}\;\left[\Gamma_{0}/\left({\mathrm{r̅}}_{0}\sqrt{\nu }\right)\right]}{\left(\varepsilon_{1}+\varepsilon_{2}\right){\mathrm{r̅}}_{0}}$$

is the dimensionless function of the TD film parameters with

$$1/{\mathrm{r̅}}_{0}=\left(\varepsilon_{1}+\varepsilon_{2}\right)a_{B}/\left(\varepsilon d\right)=a_{B}/\left(2\pi \alpha_{2D}\right)$$

For *d* → 0, the *F* function power series expansion at infinity does not contain even-degree terms, which makes the first-order series expansion term quite a good approximation when *d* is small enough. Then eq 6 results in

$$F=2\sqrt{\nu }/\left(\varepsilon_{1}+\varepsilon_{2}\right)$$

for any *ε* > *ε*_1,2_, and eq 5 subject to Γ/Γ_0_ = 1 yields the constraint

$$t=2\sqrt{3\nu \left[2\sqrt{\nu }/\left(\varepsilon_{1}+\varepsilon_{2}\right)-\nu \right]}/\pi$$

in the (ν, *t*) two-coordinate space. This is the ’melting curve’ to divide the (ν, *t*) plane into the regions of the solid phase formed by the electron superlattice and conventional liquid phase of the free electron system. A simple extreme value analysis reveals the only point of maximum for this curve, $\nu_{0}=2.25/{\left(\varepsilon_{1}+\varepsilon_{2}\right)}^{2}$ and $t_{0}=4.5\pi^{-1}/{\left(\varepsilon_{1}+\varepsilon_{2}\right)}^{2}$ , in the square-root domain $0\leq \nu \leq 4/{\left(\varepsilon_{1}+\varepsilon_{2}\right)}^{2}$ which with the 0 ≤ *t* < *t*_0_ condition encloses the electron Wigner crystal phase. For example, for air (*ε*_1_ = 1)/MgO (*ε*_2_ = 3) superstrate/substrate atomically thin (*d* → 0) TD systems, *n*_0_ ≈ 7 × 10^14^cm^–2^, *T*_0_ ≈ 6110 K and *n* ≤ 1.25 × 10^15^cm^–2^. For *ε*_1_ = *ε*_2_ = 1 the curve turns into that of PF model to enclose the region 0 ≤ ν ≤ 1, 0 ≤ *t* ≲ 0.4 of the Wigner crystal phase for 2D electron system free-standing in air.^7^

In the most general case of the ultrathin TD films of finite-thickness, eq 5 yields the melting surface in the (*d*, ν, *t*) three-coordinate space

7 $$t=\frac{2}{\pi }\sqrt{3\nu \left[F\left(d,\varepsilon ,\varepsilon_{1,2}\right)-\nu \right]}$$

This turns into the PF melting curve when projected on the *d* = 0 plane with *ε*_1_ = *ε*_2_ = 1. In the opposite limit, raising *d* makes the square-root argument negative,^67^ and the Wigner crystal phase is rendered impossible.

The features described above for the TD film melting surfaces can be seen in Figures 2 and 3, obtained numerically from eq 7. Figure 2 shows the curves calculated for fixed *d* with *ε*_1_ = 1, *ε* = 9 and *ε*_2_ = 3 (see Figure 1) corresponding to the air/TiN/MgO TD system.^59^ The free-standing (*ε*_1_ = *ε*_2_ = 1) zero-*d* PF model curve is shown as well (true 2D case). The Wigner solid phases are bounded from the top by their respective melting curves and shaded accordingly. They can be seen to contract significantly not only with increasing *d* but also for atomically thin films deposited on a dielectric substrate as compared to the ideal 2D case.

**Figure 2:**
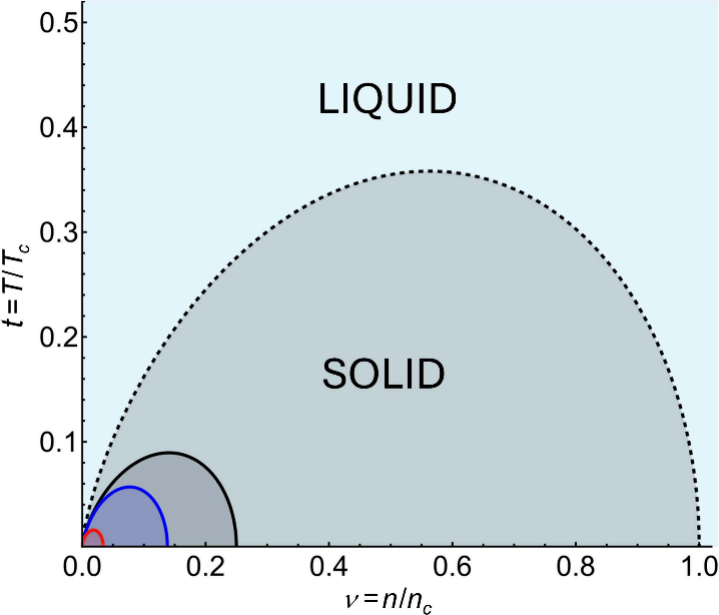
Solid–liquid phase diagrams for TD films as compared to 2D free-standing PF model (black dashed line). Red, blue, and black lines enclose Wigner solid phases (shaded accordingly) for air­(*ε*_1_ = 1)/TiN­(*ε* = 9)/MgO­(*ε*_2_ = 3) TD systems of *d* = 1 nm, 0.1 nm, and *d* → 0, respectively, as given by eq 7.

**Figure 3:**
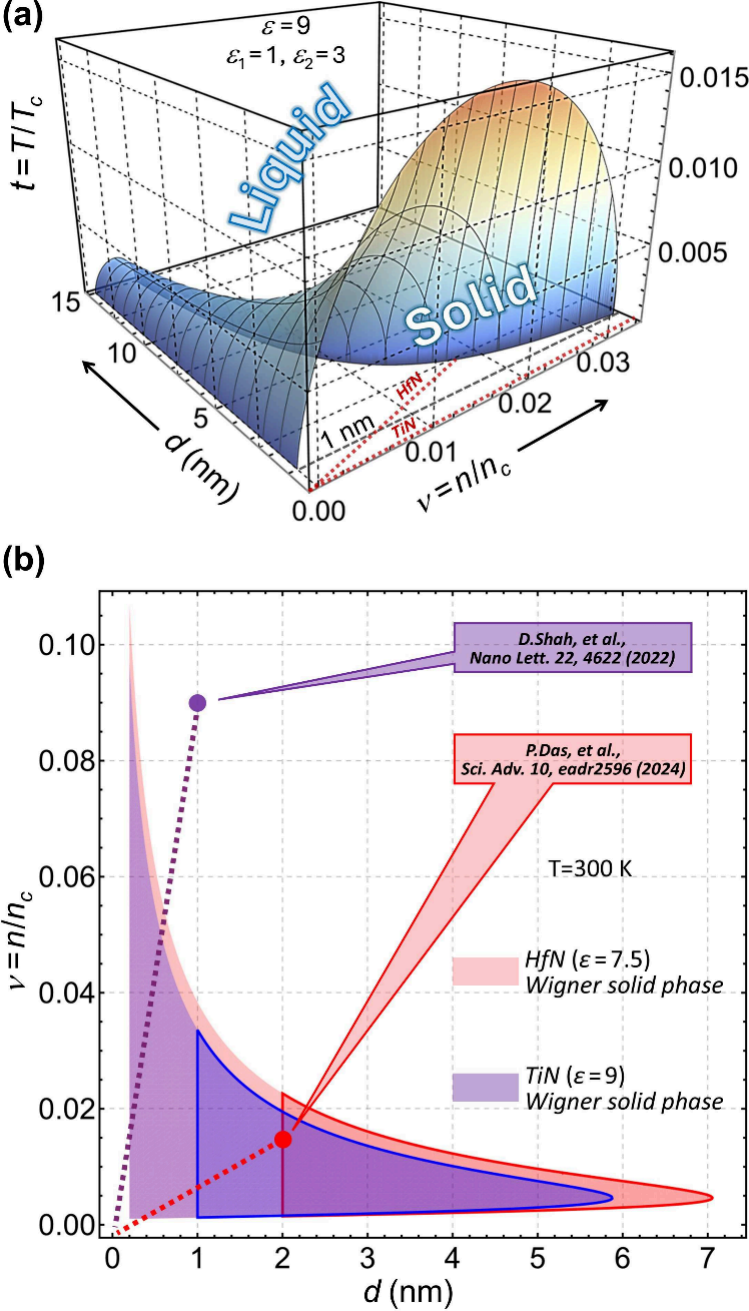
(a) Melting surface given by eq 7 for air/TiN/MgO film. (b) Horizontal crosscut of (a) showing the room-*T* Wigner crystal phase (violet) and same for air/HfN/MgO film (pink). In (a) and (b), the linear thickness dependences of the 2D electron density normalized are traced down to zero by dotted lines. Solid lines in (b) confine the areas of thicknesses achieved experimentally, and dots with callouts mark the lowest electron densities reported (4.5 × 10^14^ cm^–2^ and 7.4 × 10^13^ cm^–2^ for 1 nm thick TiN and 2 nm thick HfN films, respectively).

Figure 3(a) shows the melting surface for the air/TiN/MgO TD system in the (*d*, ν, *t*) three-coordinate space. The solid phase expands drastically as *d* → 0 while still taking just a fraction of the parameter space of the free-standing (true 2D) PF model (cf. Figure 2). The range of parameters for the room-*T* Wigner crystal phase in TD plasmonic film systems can be seen in Figure 3(b). Shown in violet and pink there are the horizontal *T* = 300 K crosscuts of the air/TiN/MgO and air/HfN/MgO (*ε* = 7.5) melting surfaces. The thick solid lines confine the parameter ranges with film thicknesses previously achieved experimentally.^59,60^ The dotted lines, in both Figure 3(a) and Figure 3(b), trace the linear *n*(*d*) dependences *n*_2*D*_ = *n*_3*D*_ *d* (in units of *n*_*c*_) for *d* small enough. The points with callouts in (b) indicate the lowest *n* measured: 4.5 × 10^14^cm^–2^ for 1 nm thick TiN film^59^ (estimated from plasma frequency) and 7.4 × 10^13^cm^–2^ for 2 nm thick HfN film^60^ (obtained from direct transport measurements). As can be seen in (b), the electron subsystem of the 2 nm thick HfN film is expected to be in the stable Wigner solid state as the electron lattice correlation energy of *k*_*B*_ *T*_*corr*_ ≈ 67 meV, given by eq 7 for this case, exceeds greatly typical room-*T* thermal fluctuation energies in 2D materials (≲10 meV, see ref 68). Due to the electrostatic repulsion spherical isotropy one should expect such a Wigner solid to be composed of the two interlocked commensurate triangular electron lattices located at the top and bottom interfaces, effectively resulting in a bilayer Wigner crystal. Thus, the plasmonic breakdown and MIT in the ultrathin finite-thickness air/HfN/MgO film system at room *T* are unavoidable just as observed.^60^ A tiny increase of *d* causes *n* (or ν) to rapidly increase, in which case the electron subsystem leaves the solid phase region, as Figures 3(a) and Figure 3(b) show, and melts out to restore the material plasmonic properties. Clearly, the effect is reversible and can be controlled both by the variation of *T* and by the electrostatic electron doping for the 2 nm thick air/HfN/MgO film system, in particular, where the drastic resistivity changes opposite to those expected of the free electron model were already observed in the *T* dependent electron transport measurements.^60^ Figure 3(b) also shows that due to higher *n* the plasmonic breakdown effect cannot occur in the air/TiN/MgO TD system of *d* ≳ 1 nm, in agreement with experiment as well.^59^ However, removing just a few monolayers of TiN could shift the electron subsystem out of the higher-*n* liquid phase region of the parameter space down to the appropriate reduced-*n* Wigner crystallization region (violet dotted line). Once it is there, the increase of both *T* and *n* (by electrostatic doping) can melt such an electron Wigner solid reversibly as Figure 3 shows.

Signatures of electron Wigner crystallization in semiconductor TMDC monolayers were recently observed indirectly in zero magnetic field by monitoring an extra exciton photoluminescence resonance interpreted as being due to the exciton Umklapp scattering by the 2D electron lattice formed at *T* ≲ 10 K.^28^ The effect was reported for *n* ∼ 10^11^cm^–2^, or ν = *n*/*n*_*c*_ ≈ 2 × 10^–5^ in terms of our theory. For such small ν, the *d*-dependence in eq 6 cancels out completely and only the substrate-superstrate dielectric factor remains for the 2D interface of *h*-BN material the TMDC monolayer is embedded in. With *ε*_1_ = *ε*_2_ ≈ 5.87 for bulk *h*-BN,^17,69^ eq 7 takes the form

$$t=T/T_{c}\approx 2\sqrt{6\nu^{3/2}/\left(\epsilon_{1}+\epsilon_{2}\right)}/\pi \approx 0.5\nu^{3/4}$$

to locate monolayer TMDCs at the very bottom left corner on the ν-axis in Figure 3 (a) where the Wigner crystal phase is bounded by *T* ≲ 10K, or at the very bottom below the shaded areas in Figure 3 (b) where no room-*T* crystal phase exists. Earlier zero-field 2D *p*-doped GaAs/AlGaAs experiments (*ε*_1_ = *ε*_2_ ≈ 12.5) fall into that region as well due to even lower $∼10^{10}\;{\mathrm{cm}}^{-2}$ carrier densities,^70^ yielding *T* ≲ 1 K for the upper bound of the crystal phase, just as was observed experimentally. In sharp contrast to zero-*T* theory predictions,^71^ at very low densities electrostatic repulsion tends to zero while kinetic energy per particle remains finite due to quantum fluctuations, whereby the potential-to-kinetic energy ratio Γ drops down necessitating lower *T* for crystallization (see ref 67 for details).

As evident from the examples of HfN and TiN ultrathin films discussed above, in order to reach the conditions required for the formation of an electron Wigner crystal, one should start with films of metals with relatively low volumetric free carrier density *n*_3*D*_, and progressively reduce their thickness. Thus, utilizing semimetals instead of conventional metals is more feasible for this purpose. Among plasmonic semimetals such as transition metal nitrides, bulk HfN is known to have *n*_3*D*_ about an order of magnitude less than bulk TiN. Since *n*_2*D*_ = *n*_3*D*_ *d* for sufficiently small thickness *d*, electron crystallization is predicted for the 2 nm thick HfN film. However, it is not expected for same thickness TiN—thinner TiN films are required to achieve electron crystallization. Moreover, the effect depends crucially on substrate and superstrate dielectric materials, which is often ignored by standard 2D theories, as well as on the in-plane background dielectric constant of the plasmonic film itself. The film thickness sufficient for electrons to crystallize can be estimated from the interelectron distance domain of the logarithmic part of the KR interaction potential in eq 3. Using $\left\langle \rho \right\rangle =1/\sqrt{\pi n_{2D}}$ , it can be formulated as

$$\left(\varepsilon_{1}+\varepsilon_{2}\right)/\left(\varepsilon \sqrt{\pi n_{2D}}\right)≲d≲1/\sqrt{\pi n_{2D}}$$

where the left boundary sets up the onset of the true 2D regime. This gives a rough estimate *d* ≲ 2 nm for the TD plasmonic films with *n*_2*D*_ ∼ 10^13^cm^–2^, which is close to the HfN case. Due to its universality, when written as

$$d/\left(2\pi \alpha_{2D}\sqrt{\pi n_{2D}}\right)≲d≲1/\sqrt{\pi n_{2D}}$$

this condition can also be applied to thin layers of organic molecular conductors,^15^ where α_2*D*_ and *n*_2*D*_ should both be treated as in-plane tensors. This points to the significance of the in-plane alignment of organometallic molecular clusters for strongly correlated electron quantum phase transitions in these quasi-2D systems. Finally, the same condition can also be formulated as

$$(\varepsilon_{1}+\varepsilon_{2})/(\varepsilon k)≲d≲1/k$$

for interface light scattering by TD plasmonic films, where (due to the momentum conservation and uncertainty principle)

$$k=\sin\theta \sqrt{\varepsilon_{1}}\omega /c∼1/\left\langle \rho \right\rangle$$

is an average plasma mode momentum excited by the light of frequency ω incident at an angle θ from dielectric medium of permittivity *ε*_1_; see Figure 1(a). In this case, one can see that the restriction on *d* can be greatly relaxed by decreasing θ, to give rise to pronounced linear and nonlinear nonlocal optical effects.^56,58,61^

To conclude, metallic TD materials offer a new approach to explore strong electronic correlations in quantum systems. Contrary to 2D semiconductors and semimetals, whose electron density is inherently rather low, for metallic TD systems the decrease of surface electron density with thickness reduction drives their electron subsystem into the crystal phase from the opposite (high electron density) side of the electron Wigner crystallization phase diagram—the region of the parameter space that has yet to be fully explored. The screening in TD metals and semimetals is greatly reduced as compared to their bulk counterparts. Metal-dielectric interface barriers are high enough ( ∼3 eV) for electron spill-out distances not to exceed just a few fractions of angstrom.^72^ In artificial 2D superlattices including moiré systems, the in-plane transport and associated Wigner crystal melting are suppressed due to a large effective mass, and even tiny imperfections can lead to irreversible disorder-related Anderson localization. In contrast, plasmonic TD materials such as TMNs are less sensitive to imperfections and so are more suitable for Wigner crystal formation.^60^ As a test, an in-plane static magnetic field can be used to reduce the number of electron translational degrees of freedom from two (in-plane motion) to one (in-plane motion along the magnetic field direction) and thus to change the Wigner crystallization picture while leaving the Anderson localization process intact. A variety of TMNs (TiN, ZrN, HfN, etc.), their ability to grow as high-quality ultrathin epitaxial films with controlled interfacial strain,^73^ and their electron density sensitivity to material parameters, provide a rich playground for the realization of strongly correlated electron systems in different regimes.^50^ Exploring the electron Wigner crystal feasibility with metallic TD materials at room *T* in zero magnetic field represents an entirely new direction in the research area of strongly correlated electron systems. It is expected to bring critical fundamental insights into the physics of strongly correlated phenomena to enable a new generation of tunable, reconfigurable and multifunctional devices for nanophotonics, optoelectronics, and advanced quantum technologies.

## Supplementary Material



## Notes

Detailed calculations and analysis of the mean electron kinetic energy per particle and generalized PF ratio temperature dependence for TD material systems (PDF)
