# Supplementary material for: Crystallization of the Transdimensional Electron Liquid
Source: Nano Lett. 2026 Mar 10;26(11):3649–55. doi: 10.1021/acs.nanolett.5c03894 (PMC13022890; doi:10.1021/acs.nanolett.5c03894)
Supplement: Supplementary file 1 [file nl5c03894_si_001.pdf]

Supporting Information for: Crystallization of the Transdimensional Electron Liquid

Igor V. Bondarev,[*,†] Alexandra Boltasseva,[‡,¶] Jacob B. Khurgin,[§] and Vladimir M. Shalaev[‡]

†*Department of Mathematics & Physics, North Carolina Central University, Durham, NC 27707, USA*

‡*Elmore Family School of Electrical & Computer Engineering, Purdue Quantum Science & Engineering Institute, and Birck Nanotechnology Center, West Lafayette, IN 47907, USA*

¶*School of Materials Engineering, Purdue University, West Lafayette, IN 47907, USA*

§*Department of Electrical & Computer Engineering, Whiting School of Engineering, Johns Hopkins University, Baltimore, MD 21218, USA*

E-mail: ibondarev@nccu.edu

Abstract

Here we provide the technicalities of the analytical calculations for the mean number of particles and mean kinetic energy per particle for free electrons in transdimensional (TD) material systems. Detailed discussion supported with illustrations is also provided to include the domain of the melting surface equation in the (d, ν, t) three-coordinate space and the generalized PF ratio temperature dependence of the TD systems.

Introduction

For optically dense planar nanostructures in the transdimensional (TD) regime, the electrostatic interaction potential of charge carriers confined is stronger than that in a homogeneous medium with the same dielectric permittivity due to the increased field contribution from outside dielectric environment with lower dielectric constant.[1,2] This interaction is associated with the Keldysh-Rytova (KR) electrostatic interaction potential,[3] in which the thickness d represents the size of the vertical electron confinement region in optically dense ultrathin planar systems, while the vertical coordinate (z-coordinate) dependence is gone. This corresponds to the effective dimensionality reduction from 3D to 2D, both in the coordinate space and in the momentum (reciprocal) space. Therefore, all sums and integrals over the momentum space presented below are 2D, and are calculated using the standard statistical physics ansatz[4]

$$\sum_{\mathbf{k}\,\in\,1st\,B.Z.}\cdots=\frac{S}{(2\pi)^2}\int d\mathbf{k}\cdots=\frac{S}{(2\pi)^2}2\pi\int dkk\cdots=\frac{S}{2\pi}\int_0^\infty d\epsilon\frac{k}{d\epsilon/dk}\cdots\,, \tag{1}$$

where summation is over the first Brillouin zone of an in-plane isotropic 2D electron system of surface area S.

Mean kinetic energy per particle at low and moderate temperatures

This is the quantum degeneracy regime of the electron gas. Due to high Fermi temperatures T_F ($\sim 10^5$ K) of typical metals, this regime spreads from the absolute zero upwards to exceed the room temperature of ~ 300 K by two to three orders of magnitude. With Eq. (1), the

mean kinetic energy per particle takes the following form

$$\langle K\rangle = \frac{2}{\langle N\rangle}\sum_{\mathbf{k}\,\in\,1st\,B.Z.}\epsilon(\mathbf{k})n_F(\mathbf{k}) = \frac{1}{\pi n}\int_0^\infty d\epsilon\frac{k\epsilon}{d\epsilon/dk}n_F(\epsilon)\,. \tag{2}$$

Here,

$$\langle N\rangle = 2\sum_{\mathbf{k}} n_F(\mathbf{k}) \tag{3}$$

is the mean number of particles (electrons) in the system (factor of 2 is to account for the electron spin degeneracy),

$$n_F(\mathbf{k}) = \frac{1}{e^{\beta[\epsilon(\mathbf{k})-\mu]}+1}, \quad \beta = \frac{1}{k_B T} \tag{4}$$

is the Fermi-particle distribution function with chemical potential μ to represent the many-particle system of quasi-free electrons with in-plane quasimomentum $\mathbf{k}$, effective mass m, and kinetic energy

$$\epsilon(\mathbf{k}) = \frac{\hbar^2 k^2}{2m}, \tag{5}$$

and

$$n = \frac{\langle N\rangle}{S} \tag{6}$$

is the electron surface density of the system. Note that the quasi-free electron (*aka* ideal electron gas) approximation is totally legitimate for degenerate electron gas systems and works the better the greater the system density is.[4]

Using the ansatz (1) together with Eqs. (4) and (5) in Eq. (3) gives

$$\langle N\rangle = 2\frac{S}{(2\pi)^2}2\pi\frac{m}{\hbar^2}\int_0^\infty\frac{d\epsilon}{e^{\beta(\epsilon-\mu)}+1}, \tag{7}$$

which after the substitution of variables

$$z = \beta(\epsilon-\mu) \tag{8}$$

becomes

$$\langle N\rangle = 2\frac{S}{(2\pi)^2}2\pi\frac{m}{\hbar^2}\frac{1}{\beta}\int_{-\beta\mu}^{\infty}\frac{dz}{e^z+1} = S\frac{m}{\pi\hbar^2\beta}\left(\int_{-\beta\mu}^{0}\frac{dz}{e^z+1}+\int_{0}^{\infty}\frac{dz}{e^z+1}\right). \tag{9}$$

Using the identity

$$\frac{1}{e^z+1} = 1-\frac{1}{e^{-z}+1} \tag{10}$$

in the first integral of Eq. (9), one further obtains the following for the parenthesized expression above

$$\begin{aligned}\int_{-\beta\mu}^{0}dz-\int_{-\beta\mu}^{0}\frac{dz}{e^{-z}+1}+\int_{0}^{\infty}\frac{dz}{e^z+1} &= \beta\mu-\int_{0}^{\beta\mu}\frac{dz}{e^z+1}+\int_{0}^{\infty}\frac{dz}{e^z+1}\\ &\approx \beta\mu-\int_{0}^{\infty}\frac{dz}{e^z+1}+\int_{0}^{\infty}\frac{dz}{e^z+1} = \beta\mu\,.\end{aligned} \tag{11}$$

Here, the replacement $\beta\mu\to\infty$ in the last step is an approximation consistent with T being less than $T_F\sim 10^5$ K, which amounts to neglecting exponentially small terms in asymptotic series expansions of Eqs. (2), (3) and such.[4]

Plugging Eq. (11) into Eq. (9) leads to

$$\langle N\rangle = S\frac{m\mu}{\pi\hbar^2}\,. \tag{12}$$

This is the final result for the mean number of particles (electrons) $\langle N\rangle$ in the system defined initially by Eq. (3), yielding also

$$n = \frac{m\mu}{\pi\hbar^2}, \tag{13}$$

as per Eq. (6), and

$$\mu = \frac{\pi n\hbar^2}{m}, \tag{14}$$

accordingly.

To calculate the mean kinetic energy per particle $\langle K\rangle$, one starts from Eq. (2) to obtain

after plugging Eq. (5) in it

$$\langle K\rangle = \frac{1}{\pi n}\frac{m}{\hbar^2}\int_0^\infty \frac{d\epsilon\epsilon}{e^{\beta(\epsilon-\mu)}+1}, \tag{15}$$

which after the substitution (8) takes the form

$$\langle K\rangle = \frac{1}{\pi n}\frac{m}{\hbar^2}\frac{1}{\beta}\int_{-\beta\mu}^\infty dz\,\frac{\mu+z/\beta}{e^z+1} = \frac{m}{\pi n\hbar^2\beta}\left(\int_{-\beta\mu}^0 dz\,\frac{\mu+z/\beta}{e^z+1}+\int_0^\infty dz\,\frac{\mu+z/\beta}{e^z+1}\right). \tag{16}$$

Here, as before, the parenthesized expression can be transformed using Eq. (10) to obtain

$$\begin{aligned}
&\int_{-\beta\mu}^0 dz\Big(\mu+\frac{z}{\beta}\Big)-\int_{-\beta\mu}^0 dz\,\frac{\mu+z/\beta}{e^{-z}+1}+\int_0^\infty dz\,\frac{\mu+z/\beta}{e^z+1}\\
&=\int_0^{\beta\mu} dz\Big(\mu-\frac{z}{\beta}\Big)-\int_0^{\beta\mu} dz\,\frac{\mu-z/\beta}{e^z+1}+\int_0^\infty dz\,\frac{\mu+z/\beta}{e^z+1}\\
&\approx\frac{\beta\mu^2}{2}-\int_0^\infty dz\,\frac{\mu-z/\beta}{e^z+1}+\int_0^\infty dz\,\frac{\mu+z/\beta}{e^z+1}\\
&=\frac{2}{\beta}\Big[\Big(\frac{\beta\mu}{2}\Big)^2+\int_0^\infty dz\,\frac{z}{e^z+1}\Big]=\frac{2}{\beta}\Big[\Big(\frac{\beta\mu}{2}\Big)^2+\frac{\pi^2}{12}\Big],
\end{aligned} \tag{17}$$

where to calculate the remaining integral in the last step, the following analytical formula in terms of Bernoulli numbers is used[4]

$$\int_0^\infty dz\frac{z^{2n-1}}{e^z+1} = \frac{2^{2n-1}-1}{2n}\pi^{2n}B_n,\quad B_1=\frac{1}{6},\ B_2=\frac{1}{30},\ B_3=\frac{1}{42},\ B_4=\frac{1}{30},\ \cdots$$

Plugging Eq. (17) into Eq. (16) and eliminating μ by using Eq. (14), one obtains the following final form of Eq. (2) defining the mean kinetic energy per particle in the system

$$\langle K\rangle = \frac{\pi n\hbar^2}{2m}\left[1+\frac{1}{3}\Big(\frac{m}{n\hbar^2\beta}\Big)^2\right]. \tag{18}$$

Mean kinetic energy per particle at high temperatures

The high-temperature regime is used in the main text with the only purpose to introduce the T_c scaling unit. This is the classical regime, for which the classical energy equipartition

theorem says that the mean kinetic energy per particle $\langle K\rangle$ in a many-particle system of weakly interacting particles is equal to $k_BT/2$ multiplied by the number of degrees of freedom of the particles in the system, thus yielding $\langle K\rangle = k_BT$ in our case. Below, for completeness of our analysis, this is demonstrated by direct analytical calculations.

In the high-T regime, the Fermi-particle distribution function (4) becomes

$$n_F(\mathbf{k}) = \frac{1}{e^{\beta[\epsilon(\mathbf{k})-\mu]}+1} \approx e^{\beta\mu} e^{-\beta\epsilon(\mathbf{k})}, \tag{19}$$

and Eq. (2) in view of Eqs. (3) and (1) takes the form

$$\begin{aligned}\langle K\rangle = \frac{\sum_{\mathbf{k}} \epsilon(\mathbf{k}) e^{-\beta\epsilon(\mathbf{k})}}{\sum_{\mathbf{k}} e^{-\beta\epsilon(\mathbf{k})}} &= -\frac{\partial}{\partial\beta} \ln \sum_{\mathbf{k}} e^{-\beta\epsilon(\mathbf{k})} = -\frac{\partial}{\partial\beta} \ln \frac{Sm}{2\pi\hbar^2} \int_0^\infty d\epsilon \, e^{-\beta\epsilon} \\ &= -\frac{\partial}{\partial\beta} \ln \frac{Sm}{2\pi\hbar^2\beta} = \frac{1}{\beta}\end{aligned} \tag{20}$$

as expected.

Domain of definition of the melting surface equation

The melting surface equation presented in Eq. (7) of the main text has the form

$$t = \frac{2}{\pi} \sqrt{3\nu\big[F(d,\varepsilon,\varepsilon_{1,2}) - \nu\big]}, \tag{21}$$

where $\nu = n/n_c$, $t = T/T_c$, and $F(d,\varepsilon,\varepsilon_{1,2})$ is the dimensionless function of actual TD film parameters. Its projection on the $d = 0$-plane of the (d,ν,t) three-coordinate space takes the form

$$t = \frac{2}{\pi} \sqrt{3\nu\Big(\frac{2\sqrt{\nu}}{\varepsilon_1 + \varepsilon_2} - \nu\Big)}, \tag{22}$$

which turns into the PF melting curve

$$t = \frac{2}{\pi} \sqrt{3\nu\big(\sqrt{\nu} - \nu\big)} \tag{23}$$

when $\varepsilon_1 = \varepsilon_2 = 1$.

Figure S1 shows the domain of Eq. (23). This is the light-red shaded area bounded by the functions $\sqrt{\nu}$ and ν from the top and bottom, respectively. Figure S2, calculated for the air($\varepsilon_1 = 1$)/TiN($\varepsilon = 9$)/MgO($\varepsilon_2 = 3$) TD system as an example, shows the domain of Eq. (21). This can be seen to be the segment of 3D space above the $F = \nu$-plane. It can be seen that for quite a broad range of d, or screening lengths $r_0 = \varepsilon d/(\varepsilon_1 + \varepsilon_2)$, there is always a solution to guarantee the Wigner solid phase in the vicinity of $\nu \gtrsim 0$, while in the vicinity of $\nu \lesssim 0.25$, or more generally $\nu \lesssim 4/(\varepsilon_1 + \varepsilon_2)^2$ representing the right boundary of the domain of Eq. (22), solutions are only possible for d low enough. These solutions are strongly dependent on the permittivities of substrate and superstrate materials and to a lesser extent on d itself. As a consequence, by choosing an appropriate substrate material it is possible to shift the right domain boundary of Eq. (22) closer to the right domain boundary $\nu = 1$ of the idealized PF model shown in Fig. S1. For example, by choosing a substrate with $\varepsilon_2 = 2$ (teflon) instead of 3, one shifts the TiN right domain boundary to $\nu = (2/3)^2 \approx 0.44$. This pushes the domain of Eq. (22) out to the right by almost twice to give $n = 0.44 n_c \approx 2.2 \times 10^{15}\,\mathrm{cm}^{-2}$ for the electron density at the right boundary of the Wigner solid phase, in which case the ultrathin TiN system with $d \lesssim 1$ nm enters (or is about to enter) the solid phase region from the right in the very same way the 2 nm-thick air/HfN/MgO TD system does as shown in the main text.

Surface electron density lowering and simultaneous shifting of the Wigner solid phase boundary towards higher electron densities due to the thickness reduction and proper choice of substrate materials, open up the opportunity to cross into the Wigner solid phase region through the higher electron density boundary $\nu \lesssim 4/(\varepsilon_1 + \varepsilon_2)^2$. This makes TD systems advantageous in studies of strong electron correlation phenomena such as Wigner crystallization at elevated temperatures. This is an advantage over 2D systems such as monolayer and quasi-monolayer semiconductor TMDC materials,[6] which are typically restricted to work at electron densities near the left boundary $\nu \gtrsim 0$ of the Wigner solid phase region and so at cryogenic temperatures as explained in the main text.

Temperature-dependent PF ratio of TD films

The generalized PF ratio presented in Eq. (5) of the main text can be rewritten as follows

$$\Gamma(d,\nu,t,\varepsilon,\varepsilon_{1,2}) = \Gamma_0 \, \frac{12\nu F(d,\varepsilon,\varepsilon_{1,2})}{12\nu^2+\pi^2 t^2}, \tag{24}$$

which in the limit of $d\to0$ takes the form

$$\Gamma(\nu,t,\varepsilon,\varepsilon_{1,2}) = \Gamma_0 \, \frac{24\nu^{3/2}}{(\epsilon_1+\epsilon_2)(12\nu^2+\pi^2 t^2)} \tag{25}$$

to give

$$\Gamma(\nu,t) = \Gamma_0 \, \frac{12\nu^{3/2}}{12\nu^2+\pi^2 t^2}$$

for the idealized 2D electron gas system free-standing in air. Notably, it can be seen from these equations that while converging to zero for $\nu\to0$ and $\nu\to+\infty$ at all non-zero T and at all T, respectively, with maximum in between at $\nu_m=\pi t/2$ raising up as T decreases, they all are divergent as $1/\sqrt{\nu}$ for $\nu\to0$ in the artificial case of the absolute zero of temperature. While the dependence of the limiting value on the path taken is not surprising for a function of two variables, in reality the electrostatic repulsion tends to zero at very low ν and kinetic energy per particle remains finite due to quantum fluctuations. Therefore, the potential-to-kinetic energy ratio must go to zero for $\nu\to0$ at all T, including $T=0\,$K. This is in sharp contrast to zero-T theory predicting more favorable crystallization conditions for $\nu\to0$,[5] which is why these predictions should not be taken for granted.

Figure S3 shows the $\Gamma(d,\nu)$ surfaces calculated from Eq. (24) for the air($\varepsilon_1=1$)/TiN($\varepsilon=9$)/MgO($\varepsilon_2=3$) TD system at $T=20$ K (dark yellow), 100 K (blue), and 300 K (green) for ν in the domain corresponding to $n\sim10^{13}\div10^{14}\,\text{cm}^{-2}$ discussed in the main text. Red plane at the bottom is the $\Gamma=1$ plane. Figure S4 shows the same surfaces in the domain of extremely low ν corresponding to electron densities $n\lesssim10^{11}\,\text{cm}^{-2}$ typical of quasi-2D semiconductor materials such as TMDC and GaAs heterostructures.[6,7] All three surfaces in Fig. S3 can be

seen to fulfil inequality $\Gamma \gtrsim 10$ for $\nu \sim 0.01$ (or $n \sim 5\times10^{13}\,\mathrm{cm}^{-2}$) and $d \lesssim 1\div3$ nm. This is more than enough to favor the electron Wigner crystallization effect at room T and below.

In Figure S4, on the contrary, only the yellow surface ($T = 20$ K) can be seen being above the $\Gamma = 1$ plane and the other two are well below. Moreover, it can be seen that by decreasing ν, or by increasing T, one makes it go below the $\Gamma = 1$ plane, too, which would lead to melting of the Wigner solid already formed. This explains the main signatures of electron Wigner crystallization previously reported for zero-magnetic field experiments both with quasi-monolayer TMDC semiconductors[6] and with p-doped GaAs/AlGaAs heterostructures.[7]

Thus, it is generally a mistake to think that by reducing the carrier density one would provide better conditions for Wigner solid formation in quasi-2D systems. As a matter of fact, this is only the case for TD metallic and semimetallic compounds whose original electron density is relatively high. By lowering it due to thickness reduction one makes the electron system enter the Wigner solid phase region ($\Gamma > 1$) from the high ν side as Fig. S3 shows. The intrinsic electron density of quasi-2D semiconductors is a few orders of magnitude lower. They are situated at the low density side ($\nu \sim 0$) of the Wigner solid phase region, where at all finite temperatures, no matter how low they are, the reduction of the carrier density generally drives the system out of the Wigner solid phase region. This can be seen in Figs. S3 and S4 as well as in the melting surface graphs presented in the main text.

References

(1) P.Cudazzo, I.V.Tokatly, and A.Rubio, Dielectric screening in two-dimensional insulators: Implications for excitonic and impurity states in graphane, Phys. Rev. B 84, 085406 (2011).

(2) J.Deslippe, M.Dipoppa, D.Prendergast, M.V.O.Moutinho, R.B.Capaz, and S.G.Louie,

Electron-hole interaction in carbon nanotubes: novel screening and exciton excitation spectra, Nano Lett. 9, 1330 (2009).

(3) L.V.Keldysh, Coulomb interaction in thin semiconductor and semimetal films, Pis'ma Zh. Eksp. Teor. Fiz. 29, 716 (1979) [Engl. translation: JETP Lett. 29, 658 (1979)]; N.S.Rytova, Screened potential of a point charge in a thin film, Moscow University Physics Bulletin 3, 30 (1967).

(4) L.D.Landau and E.M.Lifshitz, Statistical Physics, Part 1 (Course of Theoretical Physics, V.5), 3rd ed., Oxford, 1980.

(5) B.Tanatar and D.M.Ceperley, Ground state of the two-dimensional electron gas, Phys. Rev. B 39, 5050 (1989).

(6) T.Smoleński, P.E.Dolgirev, C.Kuhlenkamp, A.Popert, et al., Signatures of Wigner crystal of electrons in a monolayer semiconductor, Nature 595, 53 (2021); Y.Zhou, J.Sung, E.Brutschea, I.Esterlis, et al., Bilayer Wigner crystals in a transition metal dichalcogenide heterostructure, ibid. 595, 48 (2021).

(7) J.Yoon, C.C.Li, D.Shahar, D.C.Tsui, and M.Shayegan, Wigner crystallization and metal-insulator transition of two-dimensional holes in GaAs at $B=0$, Phys. Rev. Lett. 82, 1744 (1999).

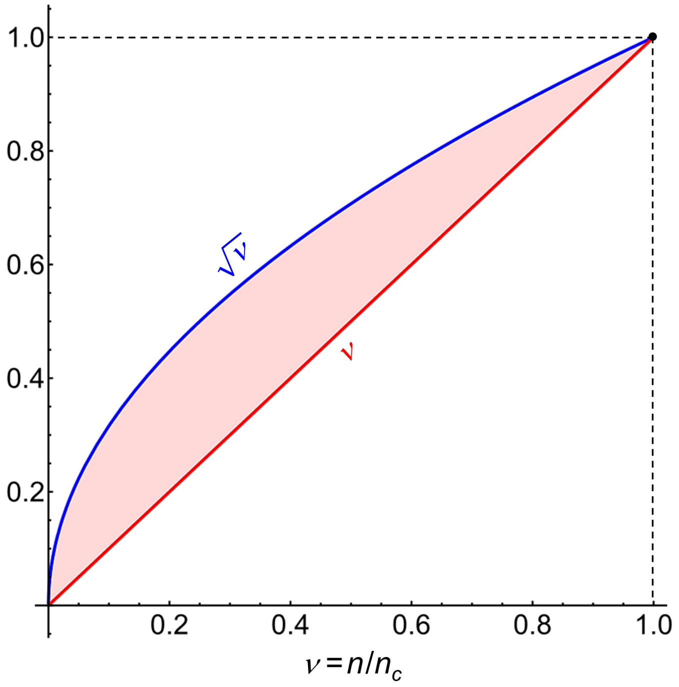

Figure S1: The domain of the PF model melting curve (23) is the light-red shaded area bounded by the functions $\sqrt{\nu}$ and ν from the top and bottom, respectively.

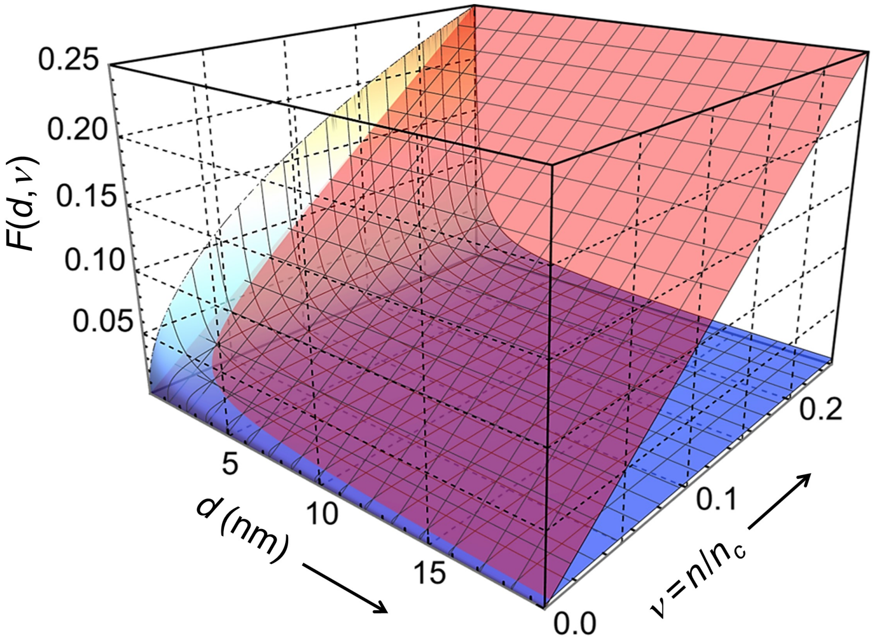

Figure S2: The domain of the general melting surface equation (21) lies above the $F = \nu$-plane (light-red) in the (d, ν, F) three-coordinate space. Shown here is the graph calculated for the air($\varepsilon_1 = 1$)/TiN($\varepsilon = 9$)/MgO($\varepsilon_2 = 3$) TD system.

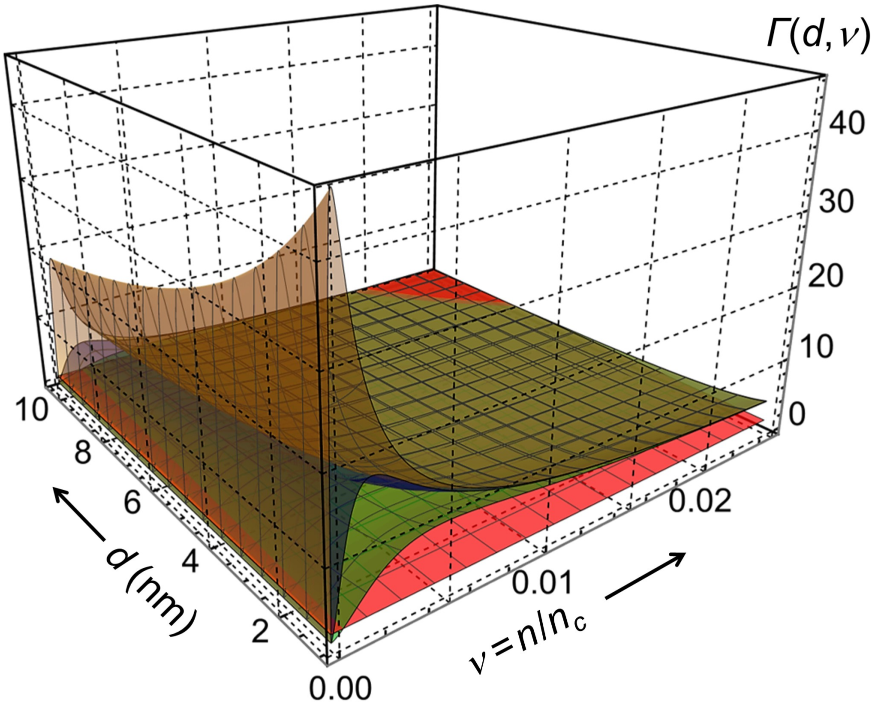

Figure S3: The $\Gamma(d,\nu)$ surfaces calculated from Eq. (24) for the air$(\varepsilon_1 = 1)$/TiN$(\varepsilon = 9)$/MgO$(\varepsilon_2 = 3)$ TD system at temperatures $T = 20$ K (dark yellow), 100 K (blue), and 300 K (green) for ν in the domain corresponding to $n \sim 10^{13} \div 10^{14}\,\mathrm{cm}^{-2}$. Red plane at the bottom is the $\Gamma = 1$ plane.

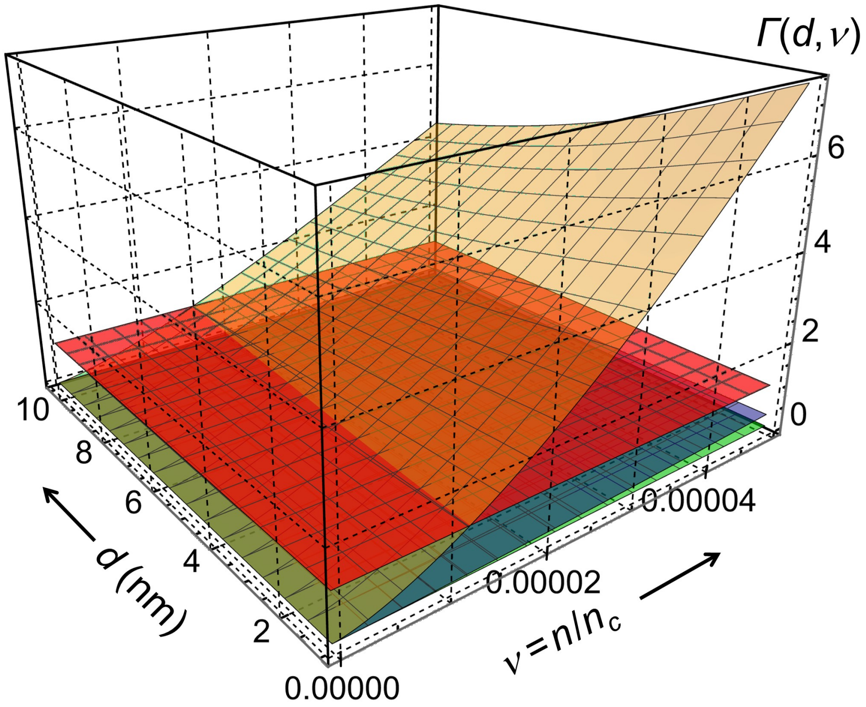

Figure S4: Same as in Fig. S3 plotted in the domain of extremely low ν corresponding to electron densities $n \sim 10^{11}\,\mathrm{cm}^{-2}$.
